# Integrating drug effects on individual cardiac ionic currents and cardiac action potentials to understand nonclinical translation to clinical ECG changes

**DOI:** 10.3389/fphar.2025.1674861

**Published:** 2025-11-10

**Authors:** Lars Johannesen, Najah Abi-Gerges, Katrina Sweat, Ana Roup, Guy Page, Claudia Alvarez Baron, Huimei Yu, Wendy W. Wu

**Affiliations:** 1 Division of Cardiology and Nephrology, Office of Cardiology, Hematology, Endocrinology and Nephrology, Office of New Drugs, Center for Drug Evaluation and Research, US Food and Drug Administration, Silver Spring, MD, United States; 2 AnaBios Corporation, San Diego, CA, United States; 3 Division of Applied Regulatory Science, Office of Clinical Pharmacology, Office of Translational Science, Center for Drug Evaluation and Research, US Food and Drug Administration, Silver Spring, MD, United States

**Keywords:** hERG, torsade, QT prolongation, Nav1.5, CaV1.2, cardiac safety, human ventricular myocytes, multi-ion channel block

## Abstract

Concomitant inhibition of the late Na^+^ current (I_NaL_) and/or the L-type Ca^2+^ current (I_CaL_) has been hypothesized to mitigate hERG block-mediated QT_C_ prolongation. This hypothesis was tested in a clinical trial using drugs selected based on available patch clamp data at the time. The results showed that hERG block-mediated QT_C_ prolongation with dofetilide was shortened by co-administration of lidocaine or mexiletine–drugs that inhibit I_NaL._ However, diltiazem, selected as the preferential I_CaL_ inhibitor, did not shorten hERG block-mediated QT_C_ prolongation by moxifloxacin. Patch clamp results can be sensitive to experimental differences across laboratories. Therefore, this study reexamined the effects of all drugs on I_NaL_, I_CaL_, and hERG current using overexpression cell lines and physiologically relevant experimental protocols aimed at producing drug-channel interaction characteristics in humans. Drug effects on ventricular action potentials (APs) from adult human trabeculae were also tested to better understand the nonclinical and clinical findings. Mexiletine and lidocaine showed similar potencies on inhibiting I_NaL_ and I_CaL_ in the prior and present patch clamp studies. Both drugs reduced dofetilide-induced AP duration (APD) prolongation, consistent with the clinical data. For diltiazem, the I_CaL_ potency and the separation between I_CaL_ and hERG potencies (I_CaL_: 1.3 µM; hERG: 8.9 µM; hERG-to-I_CaL_ ratio = 7) is much reduced comparing to the prior results (I_CaL_: 112.1 nM; hERG: 6.6 µM; ratio = 59). These new findings are consistent with diltiazem-induced APD shortening and AP triangulation caused by greater reductions in the early rather than late repolarization–a signature of multi-ion channel block. Consistent with this interpretation, nifedipine, which preferentially inhibits I_CaL_ over hERG (I_CaL_: 13.2 nM; hERG: 35 μM; ratio = 2,651) caused APD shortening without AP triangulation. Results from this study thus support the following: 1) diltiazem failed to reduce moxifloxacin-induced QT_C_ prolongation due to its concomitant hERG block at clinical exposure levels; and 2) the importance of using physiologically relevant protocols to generate ion channel pharmacology and obtaining functional recordings from myocytes to provide a better understanding of nonclinical data translation to clinical ECG signals. Data used in this manuscript, including the original electrophysiology records, may be found at: https://osf.io/69ght/.

## Introduction

The most common cause of drug-induced QT_C_ prolongation and the associated ventricular tachyarrhythmia Torsade de Pointes (TdP) is small molecule-mediated block of hERG channels that contribute to repolarize the ventricular action potential (AP) (Roden, 2004). However, TdP is reportedly rare for amiodarone, which is associated with hERG block and QT_C_ prolongation (Mattioni et al., 1989), suggesting that amiodarone’s additional electrophysiological properties mitigate proarrhythmia. Ranolazine and verapamil are also associated with hERG block and QT_C_ prolongation, but are considered to have low TdP risk due to concomitant inhibition of inward currents that contribute to cardiac APD, including I_NaL_ (Belardinelli et al., 2006) and I_CaL_ (Shimizu, 1995; Bourgonje et al., 2013). Consistent with the hypothesis that inward current inhibition can mitigate the risk posted by hERG block, administration of an I_NaL_ (Chezalviel-Guilbert et al., 1995; Belardinelli et al., 2013) or I_CaL_ inhibitor (Bourgonje et al., 2013) reduced hERG block-induced repolarization delay and TdP in animal models. Shortening of quinidine-induced QT_C_ prolongation has also been observed clinically with mexiletine, which inhibits I_NaL_ (Duff et al., 1983; Duff et al., 1987; Giardina and Wechsler, 1990). Altogether, these results have led to the Comprehensive *in vitro* Proarrhythmia Assay (CiPA) initiative aimed at developing a path of using multi-cardiac ion channel data to improve proarrhythmia risk prediction (Sager et al., 2014).

**FIGURE 1:**
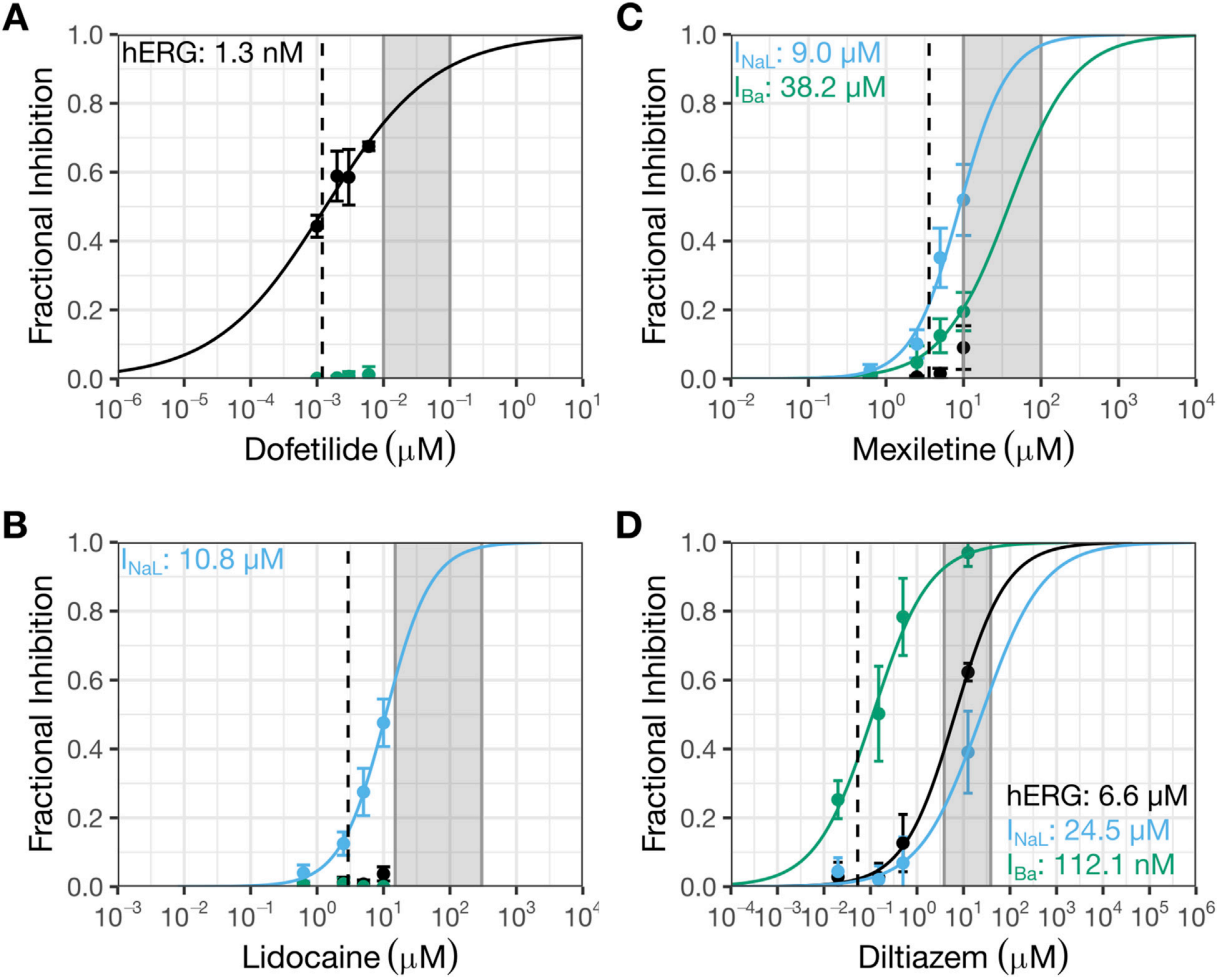
Prior patch clamp data interpreted to indicate selective inhibition of I_NaL_ by mexiletine or lidocaine, and selective block of Ca_V_1.2 channels by diltiazem. This figure shows prior patch clamp data from Crumb et al., 2016 that were used to select drug pairs and doses for testing in the clinical trial by Johannesen et al., 2016. The data were generated using manual whole cell patch clamp method at 36 °C and recombinant cell lines stably expressing hERG, Ca_V_1.2, or Na_V_1.5 channels. See Crumb et al., 2016 for details. Concentration-inhibition plots for dofetilide **(A)**, lidocaine **(B)**, mexiletine **(C)**, and diltiazem **(D)**. The Y-axis shows fractional inhibition; the X-axis, nominal drug concentrations. Error bars denote ±SE. The gray shaded region corresponds to the concentration range evaluated in the human trabeculae recordings (Table 2). The dashed line represents free clinical C_max_ from (Johannesen et al., 2016): dofetilide, 1.2 nM; lidocaine, 2.9 µM; mexiletine, 3.6 µM; and diltiazem, 53.1 nM. Free C_max_ are calculated using the following information: dofetilide (molecular weight or MW: 441.6 g/mol; percent protein binding or PB: 65%), lidocaine (MW: 234.3 g/mol; PB: 70%), mexiletine (179.26 g/mol; PB: 55%), and diltiazem (MW: 414.5 g/mol; PB: 75%). In these plots, black symbols and curves denote data for hERG; blue, I_NaL_; and green, I_Ba_ or Ba^2+^ current through Ca_V_1.2 channels.

**FIGURE 2:**
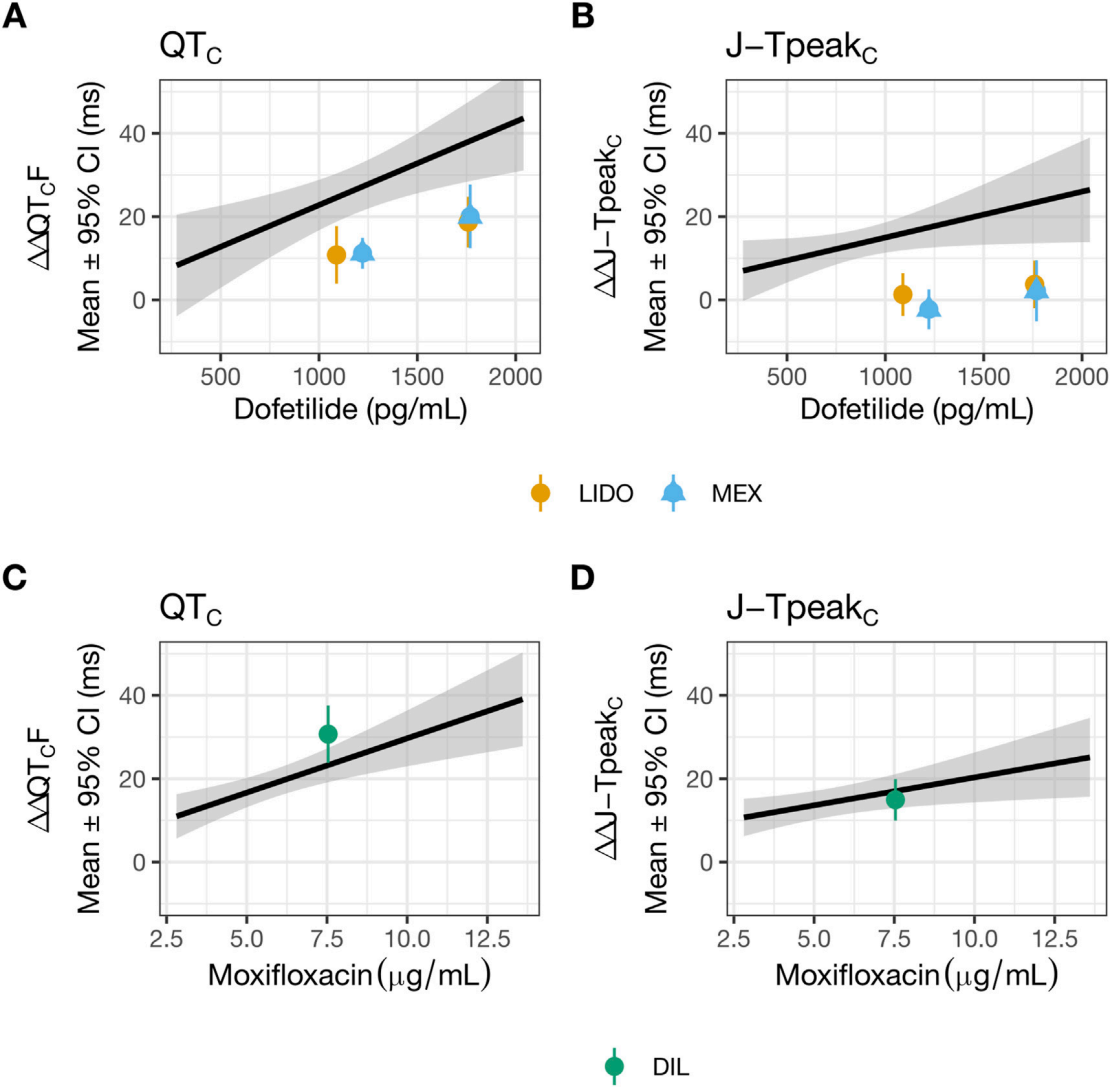
Lidocaine or mexiletine, but not diltiazem, reduced hERG block-induced QT_C_ and J-T_peakC_ prolongation. Data used to generate this figure have been published (Johannesen et al., 2016). The plasma drug concentration-dependent analysis for QT_C_ **(A,C)** and J-T_peakC_ **(B,D)** for dofetilide co-administered with lidocaine (orange) or mexiletine (blue) **(A,B)**, and for moxifloxacin co-administered with diltiazem (green) **(C,D)**. The solid line as well as shaded area reflects the fit of a linear model between drug concentration (dofetilide in panels **(A,B)** and moxifloxacin in panels **(C,D)** and placebo and baseline-corrected changes in each ECG interval. The points with error bars (95% CI) reflect the maximum change when dofetilide is combined with lidocaine or mexiletine **(A,B)**, or when moxifloxacin is combined with diltiazem **(C,D)**. LIDO, lidocaine; MEX, mexiletine; DIL, diltiazem.

Quinidine and dofetilide are preferential and selective blockers for hERG channels, respectively. Both drugs are associated with prolongation of the QT_C_ and J-T_peakC_ intervals (Johannesen et al., 2014). In contrast, multi-ion channel blockers ranolazine and verapamil are not associated with J-T_peakC_ prolongation (Johannesen et al., 2014; Vicente et al., 2019). To continue building knowledge of how to use ECG subinterval analysis to inform drug effects on multiple ion channels, a prospective clinical study was conducted using drug pairs that inhibit hERG and I_NaL_ or I_CaL_. Based on the available patch clamp data at the time (Figure 1; (Crumb et al., 2016)), dofetilide and moxifloxacin were selected as the hERG blockers, mexiletine and lidocaine as the I_NaL_ inhibitors, and diltiazem as the cardiac Ca^2+^ or Ca_V_1.2 channel blocker (Johannesen et al., 2016). The study showed that hERG block-induced QT_C_ and J-T_peakC_ prolongation were shortened by co-administration of lidocaine or mexiletine (Figures 2A,B) but not by diltiazem (Figures 2C,D). On the surface, diltiazem’s results contradict with Ca^2+^ channels’ contribution to APD and QT_C_. However, the prior results for diltiazem were obtained using Ba^2+^ as the charge carrier (Crumb et al., 2016). This substitution for Ca^2+^ is known to alter Ca_V_1.2 channel gating and modify its pharmacology in a drug-specific manner (Ren et al., 2022). Additionally, I_NaL_ recordings for lidocaine and mexiletine were performed using veratridine as the agonist (Crumb et al., 2016). Veratridine shares overlapping binding sites with lidocaine, and its use has also been shown to impact drug potencies (Wu et al., 2019). Thus, the present study reexamined the effects of all drugs in Johannesen et al., 2016 plus nifedipine on I_NaL_ (using ATX-II as the agonist), I_CaL_ (using Ca^2+^ as the charge carrier), and hERG current using overexpression cell lines. The effects of these drugs were also tested on APs recorded from adult ventricular trabeculae from organ donors to provide an integrated understanding of why diltiazem failed to reduce moxifloxacin-induced prolongation of QT_C_ and J-T_peakC_ intervals.

## Materials and methods

### Patch clamp studies of drug effects on individual cardiac ionic currents

#### Cell lines

Experiments were performed using the following stable overexpression cell lines: 1) for hERG current experiments, human embryonic kidney 293 cells (HEK293) expressing the hERG1a subunit (provided by Dr. Gail Robertson, University of Wisconsin-Madison) (Trudeau et al., 1995; 1996; Zhou et al., 1998); 2) for I_CaL_ experiments, Chinese hamster ovary (CHO) cells expressing hCa_V_1.2 α1C, β2, α2δ1 subunits (accession numbers NM_000719.4, NM_000724.2, and NM_000722.2) (Catalog #: CT6400; Charles River Laboratories, OH, USA); and 3) for I_NaL_ recordings, HEK293 cells expressing hNa_V_1.5α and β1 subunits (accession numbers XP_011532293 and NP_001028.1) (SB Drug Discovery, Glasgow, UK).

For HEK293 hERG cells, the complete growing media was Dulbecco’s Modified Eagle’s Medium (DMEM) (Thermo Fisher Scientific, 11995073) supplemented with 10% fetal bovine serum (FBS) (Gemini Bio-Products 100-106), MEM non-essential amino acids (Thermo Fisher Scientific, 11140050), and 0.25 mg/mL G418 (Thermo Fisher Scientific, 10131035). For CHO Ca_V_1.2 cells, the complete growing media was Ham’s F-12 (Thermo Fisher Scientific, 11765054) supplemented with 10% tetracycline-screened FBS (Hyclone, SH30070.03), 0.01 mg/mL blasticidin (Thermo Fisher Scientific, A1113903), 0.25 mg/mL G418, 0.25 mg/mL hygromycin B (Sigma, H0654), and 0.4 mg/mL zeocin (Thermo Fisher Scientific, R25001). For HEK293 Na_V_1.5 cells, the complete growing media was MEM (Sigma, M5650) supplemented with 10% FBS (Thermo Fisher Scientific, 10437028), 2 mM L-glutamine (Sigma, G7513), 0.6 mg/mL G418, and 4 μg/mL blasticidin.

Cells were maintained at below 90% confluence and used before passage 30 after thawing. For patch clamp recordings, cells were seeded 2–48 h before recordings onto 12 mm diameter sterilized glass coverslips (Fisher Scientific, 12–545-81P) in 35 mm Petri dishes (Corning, 430588). HEK293 hERG cells were detached using either a brief digestion with 0.25% Trypsin-EDTA (Thermo Fisher Scientific, 25200072) or TrypLE Express (Thermo Fisher Scientific, 12604013). CHO Ca_V_1.2 cells were detached using accutase (Sigma, A6964), and HEK293 Na_V_1.5 cells were detached using TrypLE Express. Detailed cell culture protocols can be found at: https://osf.io/69ght/.

#### Whole cell voltage clamp electrophysiology

Glass coverslips with attached cells were placed in a recording chamber mounted on an inverted microscope (Model: Axiovert 135TV or AX10 VertA1, Zeiss). The recording chamber was continuously perfused with external solution flowing at a rate of 1–3 mL/min. Recordings were made using borosilicate glass pipettes (BF150-86-10; Sutter Instrument, CA) pulled with a micropipette puller (P97; Sutter Instrument, CA) to 1.5–3 MΩ resistance when filled with the specified internal solutions. All experiments were conducted at near physiological temperature (37 °C ± 2 °C). Temperatures of the in-line solution heater and recording chamber were maintained with a dual channel temperature controller (TC2BIP from Cell MicroControls), and temperature of the perfusate near the recorded cells was recorded throughout the experiment with a thermistor.

For the hERG current, the external solution contained (in mM): 130 NaCl, 10 HEPES, 5KCl, 1 MgCl_2_·6H_2_O, 1 CaCl_2_·2H_2_O, and 12.5 dextrose; pH adjusted to 7.4 with 5 M NaOH. The internal solution contained (in mM): 120 K-gluconate, 20 KCl, 10 HEPES, 5 EGTA, and 1.5 MgATP; pH adjusted to 7.3 with 1 M KOH; ∼280 mOsM. The voltage command was corrected for the 15 mV liquid junction potential (calculated using pClamp10 software; Molecular Devices, CA) that resulted from using these solutions. Cells were depolarized from a holding potential of −80 mV to +40 mV for 500 ms, then ramped down to −80 mV in 100 ms (−1.2 V/s). A 100 ms hyperpolarizing step from −80 mV to −90 mV was included prior to the depolarizing step to monitor input resistance throughout the recording. This voltage protocol was repeated every 5 s. For I_CaL_, the external solution contained (in mM): 137 NaCl, 4 KCl, 1.8 CaCl_2_, 1 MgCl_2_, 10 HEPES, and 10 dextrose; pH adjusted to 7.4 with 5 M NaOH. The internal solution contained (in mM): 120 aspartic acid, 120 CsOH, 10 CsCl, 10 EGTA, 5 MgATP, 0.4 TrisGTP, and 10 HEPES; pH adjusted to 7.2 with 5 M CsOH; ∼290 mOsM. The voltage command values were corrected for the 17 mV liquid junction potential. Cells were held at −80 mV, depolarized to 0 mV for 40 ms, further depolarized to +30 mV for 200 ms, and then ramped down to −80 mV in 100 ms (−1.2 V/s). A 100 ms hyperpolarizing step from −80 to −90 mV was included prior to the first depolarizing step to monitor input resistance throughout the recording. This voltage protocol was presented every 5 s. For I_NaL_ recordings, the external solution contained (in mM): 130 NaCl, 4 CsCl, 2 CaCl_2_, 1 MgCl_2_, 10 HEPES, and 10 dextrose; pH adjusted to 7.4 with 5 M NaOH. The internal solution contained (in mM): 130 CsCl, 7 NaCl, 1 MgCl_2_, 5 EGTA, and 5 HEPES; pH adjusted to 7.2 with 5 M CsOH; ∼280 mOsM. ATX-II (150 nM) was added to the external solution to slow Na^+^ channel inactivation, thereby inducing a sustained current. Cells were first hyperpolarized from −95 mV to −120 mV for 200 ms to facilitate recovery of Na_V_1.5 channels from inactivation, then depolarized to −15 mV for 40 ms, then to +40 mV for 200 ms, then ramped down to −95 mV in 100 ms (−1.35 V/s). This voltage protocol was repeated every 10 s. The aforementioned patch clamp protocols, also available at the website for the Interdisciplinary Review Team for Cardiac Safety Studies or CS-IRT (https://www.fda.gov/about-fda/center-drug-evaluation-and-research-cder/interdisciplinary-review-team-cardiac-safety-studies-formerly-qt-irt), were designed to incorporate as many physiologically relevant features of a myocyte AP as possible and practical, consistent with ICH S7B Q&A 2.1 best practice recommendations for these assays intended to support cardiac safety interpretation (ICH, 2022). Note that Ca^2+^ was used as the charge carrier to generate I_CaL_; ATX-II was used to induce I_NaL_ as it does not bind to the transmembrane domain of Na_V_1.5 channels like veratridine does, and therefore does not compete for lidocaine binding; and Cs^+^, though not a physiological ion, was used to improve voltage control for I_CaL_ and I_NaL_ recordings. All currents were evoked at a rate lower than the physiological heart rate range as a compromise to reduce activity-dependent current rundown and allow for more complete channel deactivation. Regarding ATX-II, 150 nM has been used by the present laboratory for many years to induce I_NaL._ This concentration was chosen to overcome inconsistent effect of ATX-II that this laboratory had experienced in the past. To enable longitudinal study result comparisons, this laboratory has continued to use ATX-II at 150 nM.

**FIGURE 3:**
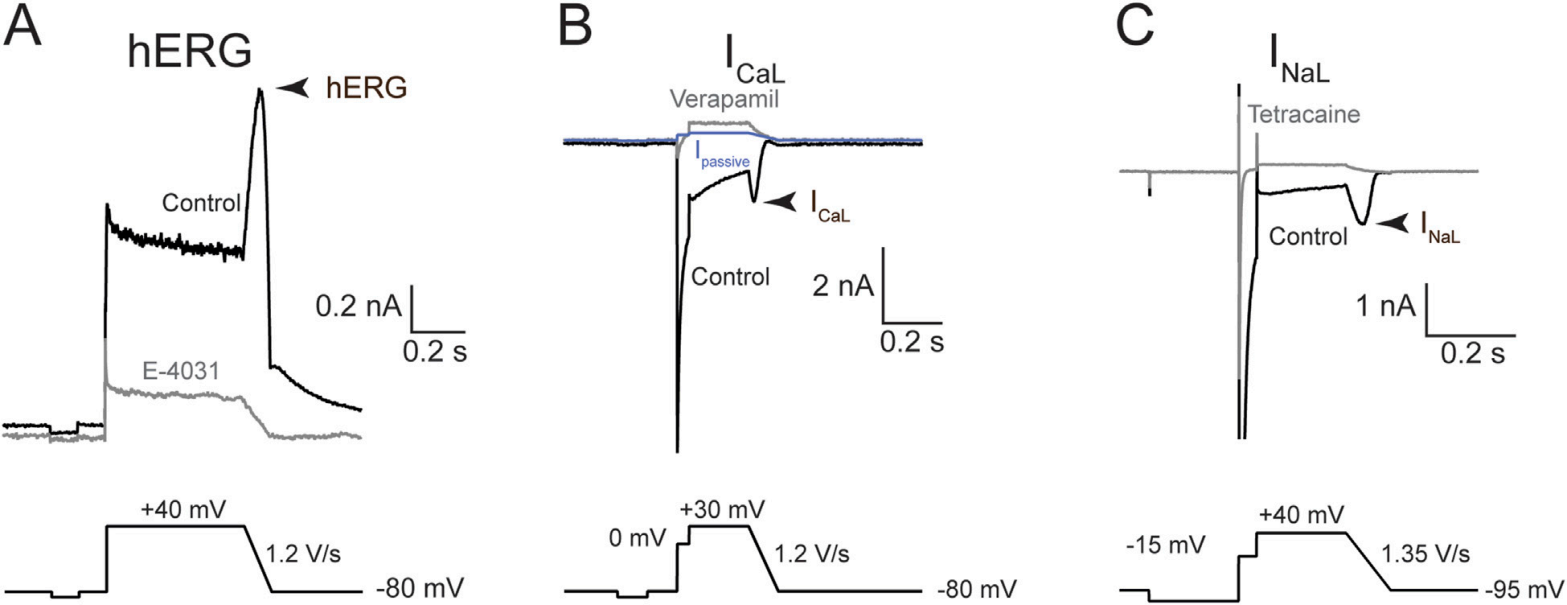
Voltage commands and representative traces for hERG, I_CaL_, and I_NaL._ Representative current traces (*top*) and volage protocols (*bottom*) used to evoke them. Each panel reflects sample traces from one recorded cell. **(A)** hERG current; **(B)** I_CaL_; and **(C)** I_NaL_. Arrowhead denotes the current region where drug effects were quantified. Black traces were obtained in the control solution; gray, after saturating concentration of a blocker to eliminate the current-of-interest. The blue trace in **(B)** is I_Passive_, calculated using the resting input resistance and Ohm’s law (see “Methods”).

These voltage protocols were continuously presented at the respective intervals throughout the recordings. Recordings were obtained first in control solution until the amplitude of the ionic current being studied reached stability, as judged by the electrophysiologist tracking current amplitude online. Then drug solution was bath-applied and recording continued until a new steady state current amplitude was reached. For I_CaL_, current rundown upon whole cell formation exhibited multiple phases. Stability for I_CaL_ experiments was thus defined as rundown reaching a steady and slower rate. Each cell was exposed to one to two concentrations of the same drug as long as membrane properties (i.e., input resistance and holding current at rest) and recording quality (new steady state current signal in the presence of the first drug concentration) remained stable. The duration of drug application varied depending on the drug, current, and concentration and was determined by the electrophysiologist using online analysis for each cell. For hERG current, drugs were applied for shorter durations for diltiazem, lidocaine and mexiletine (range: 2.4–11.8 min) and longer durations for dofetilide and nifedipine (range: 3.6–21.6 min). For I_NaL_, drugs were applied for 2.8–8.3 min. For I_CaL_, drugs were applied for 2.4–16.7 min. Figure 3 shows the voltage protocols used to elicit the currents-of-interest, with current traces from representative cells shown above the voltage protocols. Regarding data quality check, passive membrane properties (i.e., input resistance and holding current at rest) were used to indicate cell health, and recording stability (in control solution and following drug application) was used to indicate recording quality. Data were accepted if these parameters were stable.

Cells were visualized using the phase contrast method. Recordings were obtained using MultiClamp 700B amplifiers (Molecular Devices, CA). For hERG current, signals were filtered at 2.2 kHz and digitized at 5 kHz using a Digidata 1550A interface (Molecular Devices, CA), and transferred to a computer using pClamp10 software (Molecular Devices, CA). For I_CaL_ and I_NaL_, signals were filtered at 3 kHz and digitized at 10 kHz. Seal resistance was always ≥1 GΩ, and series resistance was electronically compensated at 80%. The MultiClamp 700B series resistance compensation bandwidth control replaces the “lag” control on earlier Axon amplifier series: Bandwidth = 1/(2 *π * Lag). This study used the default series resistance correction bandwidth of 1.02 kHz, which is equivalent to a lag value of 156 μs. To allow for adequate internal solution dialysis prior to actual current recording, following whole cell formation cells were given ∼2 min resting period, during which the recording and membrane properties were monitored using the “membrane test” function of the pClamp10 software.

#### Drugs and drug stock preparations for patch clamp experiments

Dofetilide, lidocaine, moxifloxacin, tetracaine, and verapamil were purchased from Sigma; diltiazem, mexiletine, nifedipine, and tetrodotoxin citrate, from Tocris. To make concentrated drug stocks, tetracaine and verapamil were dissolved in water; the rest, in DMSO. Drug stocks were kept at −20 °C for up to 6 months. On the day of use, each drug stock was thawed only once and diluted in different external solutions to prepare drug solutions for patch clamp experiments. When DMSO was used, the final concentration in the recording solution was no more than 0.1%, except for 300 µM lidocaine recordings for hERG and I_CaL_, in which the final DMSO concentration was 0.3%. In independent experiments, the effects of DMSO were assessed on hERG and I_CaL_. The average hERG current reduction was 5% in cells treated with 0.3% DMSO; I_CaL_, 16% in cells treated with 1% DMSO. These current reductions are within the expected ranges of normal rundown for these cell lines. Therefore, DMSO was not added to control solutions.

#### Data analysis for whole cell voltage clamp experiments

Current traces were analyzed using custom macros written in Igor Pro 8 (WaveMetrics, OR, USA) and pClamp10 software (Molecular Devices, CA). For the hERG current, current traces obtained in 1 µM E−4031 were first averaged then subtracted from all recorded current traces to isolate the hERG component. For I_NaL_, the current traces recorded in the presence of 300 µM tetracaine were first averaged and then subtracted from all recorded traces to isolate the Na_V_1.5 component. For I_CaL_, the current mediated by Ca_V_1.2 channels was isolated using one of two methods as described in Ren et al., 2022: 1) passive current (I_passive_) subtraction; or 2) verapamil-subtraction. For the first method, I_passive_ for each recorded current trace was calculated using the resting input resistance calculated from the current elicited by the −80 mV to −90 mV step and Ohm’s law. Since Ca_V_1.2 channels are not active at these potentials, this was considered as a passive current produced by leak currents present in the cells and through the seal between the electrode and the cell membrane. I_passive_ was then subtracted from the recorded current trace to isolate the active component, presumably mediated by Ca_V_1.2 channels. For the second method, the residual currents following application of 100 µM verapamil across multiple recorded traces were averaged and then subtracted from all recorded traces for the same cell to isolate the Ca_V_1.2 component. This method was used for cells that had endogenous nonlinear outward currents.

The potencies of tested drugs on various ionic currents were quantified by constructing concentration-inhibition plots. For all currents, fractional inhibition for each cell was calculated by first normalizing the average current amplitude of the last 10 consecutively recorded traces in drug solution to that obtained in control solution, and then subtracting this value from unity ( $1-\frac{Idrug}{Icontrol}$ ). For each current and each drug, the fractional inhibition values from all cells were pooled and plotted against the nominal drug concentrations to generate a concentration-inhibition plot. These plots were fit with the Hill equation with variable slope and minimum and maximum fractional inhibition constrained to 0 and 1, respectively, using R 4.3.2 (R Core Team, 2021) to derive block parameters and their 95% confidence interval (CI). The Hill equation is as follows: Fractional inhibition = 1/(1+ (IC_50_/[drug])^nH^). Here IC_50_ is the concentration that inhibited 50% of the current, [drug] is the drug concentration, and n_H_ is the Hill coefficient. Summary data points in the figures are shown as mean ± standard error (SE).

### Human ventricular trabeculae action potential recordings

#### Donor heart procurement

All human hearts used for this study were obtained by legal consent from organ donors in the US. Policies for donor screening and consent are the ones established by the United Network for Organ Sharing. Organizations supplying human tissues to AnaBios follow the standards and procedures established by the US Centers for Disease Control and are inspected biannually by the Department of Health and Human Services. Tissue distribution is governed by internal IRB procedures and compliance with HIPAA regulations regarding patient privacy. All organ donor transfers to AnaBios are fully traceable and periodically reviewed by US Federal authorities. Donor characteristics, heart number, and donor identifier are shown in Table 1; donor exclusion criteria implemented in this study are similar to those in Page et al., 2016 (see Table 2 of the study), and additionally included non-opioid users (Page et al., 2016).

**TABLE 1:** Donor characteristics.

Heart #	Donor identifier	Age	Sex	Ethnicity	BMI	COD	EF (%)
1	200901HHA	56	F	Caucasian	24.3	HT/Bhunt injury	59
2	200909HHA	60	F	Caucasian	25.1	CVA/ICH/Stroke	60
3	200912HHA	48	M	Hispanic	17.4	Anoxia/CVS	55
4	201116HHA	30	F	Caucasian	27.7	Anoxia	64
5	210221HHA	21	F	Caucasian	18.8	Anoxia	55
6	210408HHA	39	M	Caucasian	30.9	Anoxia/CVS	N/A^a^
7	210506HHA	55	F	Hispanic	28.3	CVA/ICH/Stroke	60
8	220315HHB	49	F	Caucasian	34.6	CVA/ICH/Stroke	68
9	220324HHA	56	F	Caucasian	26.1	CVA/ICH/Stroke	65
10	220525HHA	36	M	Caucasian	46.7	Anoxia/Seizure	62
11	220602HHB	50	M	Hispanic	27.2	HT/Blunt injury	65
12	220718HHA	51	F	Caucasian	20.3	CVA/ICH/Stroke	65
13	220808HHA	47	F	Caucasian	24.6	CVA/ICH/Stroke	65

F, female; M, male; BMI, body mass index; COD, cause of death; EF, ejection fraction; CVA, cerebrovascular accident; ICH, intracranial haemorrhage; CVS, cardiovascular; HT: head trauma; HH, human heart; HHA, the first heart received on the day; HHB, the second heart received the same day.

^a^ Organ procuremennt organization could not transplant the heart and consequently no echocardiography was performed; N/A, not available.

**TABLE 2:** Drugs and concentrations tested in adult human primary ventricular trabeculae.

Perfusion Sequence	Vehicle Control	Dofetilide 0.1 µM	Dofetilide 0.1 µM + Mexiletine 10 µM	Dofetilide 0.1 µM + Mexiletine 30 µM	Dofetilide 0.1 µM + Mexiletine 100 µM
	Vehicle Control	Dofetilide 0.1 µM	Dofetilide 0.1 µM + Lidocaine 15 µM	Dofetilide 0.1 µM + Lidocaine 30 µM	Dofetilide 0.1 µM + Lidocaine 100 µM
	Vehicle Control	Diltiazem 3.84 µM	Diltiazem 38.4 µM	Diltiazem 38.4 µM + Dofetilide 0.01 µM	Diltiazem 38.4 µM + Dofetilide 0.1 uM
	Vehicle Control	Nifedipine 0.23 µM	Nifedipine 1 µM	Nifedipine 1 µM + Dofetilide 0.01 µM	Nifedipine 1 µM + Dofetilide 0.1 µM
Recording Sequence Under Specified Perfusion Solution	25 min @ 1 Hz, then 3 min @ 2 Hz, then 3 min at 1 Hz	25 min @ 1 Hz, then 3 min @ 2 Hz, then 3 min at 1 Hz	25 min @ 1 Hz, then 3 min @ 2 Hz, then 3 min at 1 Hz	25 min @ 1 Hz, then 3 min @ 2 Hz, then 3 min at 1 Hz	25 min @ 1 Hz, then 3 min @ 2 Hz, then 3 min at 1 Hz

Some experiments were tested at both 1 and 2 Hz. Because availability cardiac ion channels is both time- and voltage-dependent, and drug block of cardiac ion channels can also be frequency-dependent, evaluating different stimulation frequencies can offer insight regarding drug effect on specific ion channels and guide further *in vitro* experiments. Data were not collected at 2 Hz for dofetilide and mexiletine or lidocaine.

#### Tissue dissection and AP recordings

The procedures of tissue dissection and sharp electrode recording were the same as Page et al., 2016. Upon arriving at the laboratory, the human heart was re-perfused with a cold (4 °C), fresh proprietary cardioplegic solution. Ventricular trabeculae were then dissected and transferred to the recording chambers.

Each single tissue was mounted into a recording chamber filled with oxygenated Tyrode’s external solution of the following composition (in mM): NaCl 136, KCl 4, MgCl_2_ 0.5, NaHCO_3_ 12, NaH_2_PO_4_ 0.35, dextrose 11.1, CaCl_2_ 1.8, HEPES 10; pH 7.4 adjusted with NaOH. The temperature of the solution was maintained at 37 °C. The flow rate was 5 mL per minute. Each tissue was allowed to equilibrate for 30–60 min with stimulation using a bipolar stimulating electrode (3 V, 3 ms) at a pacing rate of 1 Hz. High impedance borosilicate microelectrodes were prepared with a tip resistance of 10–20 MΩ when filled with 3M KCl. Upon tissue impalement, the membrane potential was allowed to stabilize. Then the tissue was continuously stimulated with the bipolar stimulating electrode using 1.5X the stimulus intensity that reliably evoked an AP, and recordings were performed using LabChart Software (ADInstruments Inc.) in continuous mode with sampling at 20 kHz.

**FIGURE 4:**
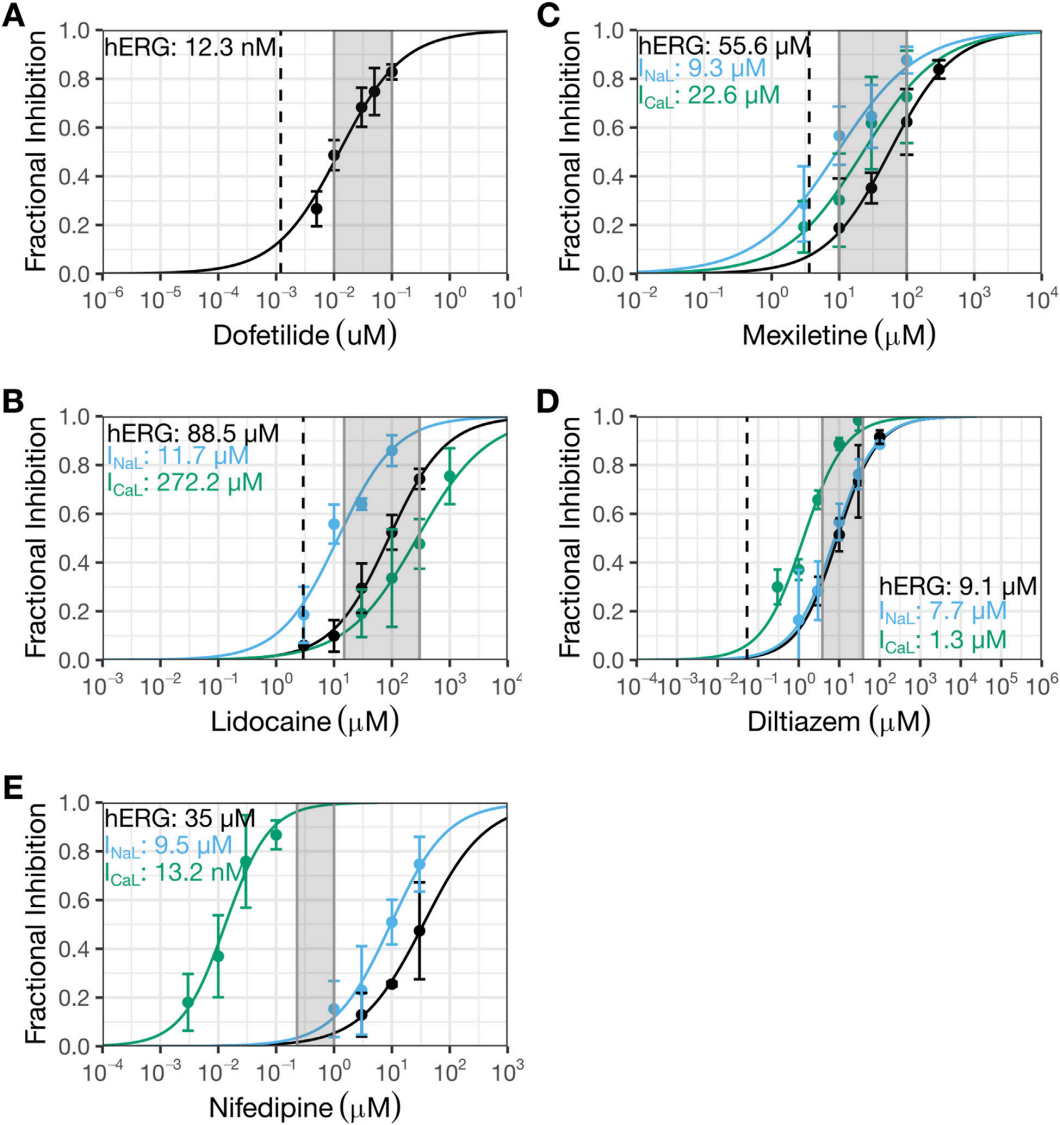
Present patch clamp data that showed diltiazem to be a multi-ion channel blocker and nifedipine to be a selective Ca_V_1.2 channel blocker. The new patch clamp experiments for dofetilide **(A)**, lidocaine **(B)**, mexiletine **(C)**, diltiazem **(D)**, and nifedipine **(E)**. The Y-axis shows fractional inhibition and the X-axis, nominal drug concentrations. Error bars denote ±SE. For Hill fits to estimate potency, fractional inhibition data for individual cells (not shown here) were used. For dofetilide **(A)**, 29 cells were recorded (4-10 cells for each concentration). For lidocaine **(B)**, 14 cells were recorded for hERG (4-9 cells for each concentration except 3 µM which was recorded in one cell), 18 cells were recorded for I_NaL_ (4-5 cells per concentration), and 11 cells were recorded for I_CaL_ (4-6 cells per concentration). For mexiletine **(C)**, nine cells were recorded for hERG (4-5 cells per concentration), 18 cells were recorded for I_NaL_ (4-5 cells per concentration), and 11 cells were recorded for I_CaL_ (5-6 cells per concentration). For diltiazem **(D)**, eight cells were recorded for hERG (4 cells per concentration), 17 cells were recorded for I_NaL_ (4 cells per concentration, except for 100 µM which was recorded in one cell), and 10 cells were recorded for I_CaL_ (4 cells per concentration). For nifedipine **(E)**, 11 cells were recorded for hERG (4-5 cells per concentration), 17 cells were recorded for I_NaL_ (4-6 cells per concentration), and 11 cells were recorded for I_CaL_ (4-6 cells per concentration). The shaded regions correspond to the regions evaluated in human action potentials (Table 2); the dashed line represents clinical concentration. HERG data for dofetilide (Alvarez Baron et al., 2022) and I_CaL_ data for diltiazem (Ren et al., 2022) had been published previously by this laboratory.

Each drug or drug pair was evaluated in a minimum of four ventricular trabeculae derived from a minimum of two donor hearts. Nominal drug concentrations chosen for testing are presented in Table 2. These concentrations were meant to cover multiples of free clinical concentrations (free C_max_) that achieved QT_C_ prolongation in Johannesen et al., 2016 and span a range on the concentration-inhibition plots based on the new patch clamp data in this manuscript and prior experiments (Table 2; Figure 4). All tissues/recordings used to generate this manuscript followed the drug application sequences, time courses, and tissue stimulation protocols as outlined in Table 2.

The following criteria were applied to exclude tissues/recordings from the dataset: 1) interruption of perfusion/oxygenation during experimentation; 2) absence or unstable APs following stimulation at baseline; 3) drug exposure time not adequate; and 4) APD at 90% repolarization <200 ms or >450 ms at baseline.

#### AP analysis

Following data acquisition, offline analysis was performed using LabChart software to measure APD at 30%, 50%, and 90% repolarization (APD_30_, APD_50_, and APD_90_, respectively). Data for each experimental condition were expressed as the mean of 30 consecutive APDs for each pacing rate tested (Table 2). The value of APD_90_ minus APD_30_ was calculated to describe AP triangulation. Beat-to-beat variability in repolarization was quantified as short-term variability (STV) from APD_90_ Poincaré plots over a period of 30 s, calculated as $\text{STV}=\frac{\sum \left|{APD90}_{n+1}-{APD90}_{n}\right|}{\left(30*\sqrt{2}\right)}$ where APD90_n_ and APD90_n+1_ are the APD_90_s for the *n*th AP and the following one, respectively. A decrease in excitability was identified when the stimulus at 1.5X intensity did not trigger an AP following complete depolarization. Effects of single drug application or co-administration of two drugs were quantified relative to the data collected during the vehicle control period. These effects are expressed as mean ± SE. Threshold values for changes over baseline are as follows: APD_30_, 12.0% and 12.1% at 1 and 2 Hz, respectively; APD_50_, 9.1% and 9.7% at 1 and 2 Hz, respectively; APD_90_, 6.9% and 7.5% at 1 and 2Hz, respectively; triangulation, 9.0% and 10.2% at 1 and 2 Hz, respectively; and STV, 102.6% and 164.3% at 1 and 2 Hz, respectively. These values have been presented in a previous study (Page et al., 2016).

#### Drugs for human trabeculae experiments

Diltiazem, nifedipine, dofetilide, sotalol, moxifloxacin, and lidocaine were purchased from Sigma (CA, USA); mexiletine, Cayman Chemical (MI, USA). Drugs were dissolved in DMSO to prepare 1000X stock solutions that were kept frozen at −20 °C for approximately 3 months. For recording, stock solutions were diluted in the Tyrode’s external solution, resulting in a working solution with 0.1% DMSO.

## Results

### Patch clamp recordings from overexpression cell lines

Figure 1 shows concentration-inhibition plots of dofetilide (Figure 1A), lidocaine (Figure 1B), mexiletine (Figure 1C), and diltiazem (Figure 1D) on hERG current, I_NaL_, and/or Ba^2+^ current from Ca_V_1.2 channels from Crumb et al., 2016 (henceforth referred to as the “prior study”). Figure 4 shows the same plots for the same currents for these four drugs plus nifedipine generated in the present study for comparisons. In each plot, the dashed line represents free clinical C_max_ from Johannesen et al., 2016; the gray shaded region corresponds to the concentration range evaluated in the human trabeculae recordings (Table 2).

The hERG IC_50_ for dofetilide is 1.3 nM (95% CI: 1–1.8 nM) (Figure 1A) in the prior study, which is ∼10X more potent than that measured in the present study (IC_50_: 12.3 nM (10.6–14.3 nM); Figure 4A). These different results led to different estimates for the degree of hERG inhibition at free C_max_: prior data suggest ∼50% hERG inhibition; present data suggest only ∼10%. For lidocaine, the prior study showed selective inhibition of I_NaL_, with an IC_50_ of 10.8 µM (9.2–12.6 µM) and minimal effects on the other currents up to 10 µM (Figure 1B). The present study confirmed stronger inhibition of I_NaL_ (IC_50_: 11.9 µM [9.5–14.8 µM]) and less effects on the other currents (hERG IC_50_: 88.5 µM (73.9–106 µM); I_CaL_ IC_50_: 272.2 µM [202.7–365.5 µM]) (Figure 4B). In the prior study, mexiletine inhibited I_NaL_ with an IC_50_ of 9.3 µM (7.5–12.6 µM) and Ba^2+^ current through Ca_V_1.2 channels with an IC_50_ of 38.2 µM (18–81.3 µM) (Figure 1C). The present study showed similar IC_50_ values for I_NaL_ and I_CaL_ as the prior study, considering patch clamp data variability that a laboratory would see if it were to retest the same drug at a later time (I_NaL_ IC_50_: 9.3 µM (6.9–12.6 µM); I_CaL_ IC_50_: 22.6 µM (15.1–33.9 µM)) (Figure 4C). For the hERG current, the highest mexiletine concentration tested in the prior study was 10 μM, which resulted in ∼10% current inhibition (Figure 1C). The present study estimated the hERG IC_50_ for mexiletine to be 55.6 µM (45.9–67.4 µM) (Figure 4C). At 10 μM, mexiletine inhibited hERG current by ∼20% - a result considered not different from the prior study. For diltiazem, the prior study showed a large separation (∼59X) between the concentration ranges required to inhibit Ba^2+^ current through the Ca_V_1.2 channels (IC_50_: 112.1 nM (77.1–163.2 nM)) and the other currents (the lowest IC_50_ across other currents was 6.6 µM for hERG (5.1–8.4 µM) (Figure 1D)). The present study showed a significantly reduced potency for I_CaL_ (IC_50_: 1.3 µM (1.1–1.6 µM); Figure 4D), resulting in a significantly reduced separation (6 to 7X) between diltiazem’s inhibition of I_CaL_ and other currents (I_NaL_ IC_50_: 7.7 µM (6.3–9.6 µM); hERG IC_50_: 9.1 µM (1.1–1.6 µM)). Based on the prior study, diltiazem would be considered a selective blocker for Ca_V_1.2 channels: when 50% of the channels were blocked, no inhibition was seen for the hERG current and I_NaL_ (Figure 1D). In contrast, the present study demonstrates diltiazem to be a multi-ion channel blocker. At 50% I_CaL_ inhibition, ∼15–20% hERG current and I_NaL_ are also inhibited.

Nifedipine was not tested in the prior study. It was included in the present study as a selective I_CaL_ inhibitor to understand how AP characteristics are affected in the human trabecular tissue experiments described below. Using the present experimental protocols, nifedipine’s IC_50_ for I_CaL_ is 13.2 nM (10–17.5 nM). The next lowest IC_50_ was for I_NaL_ at 9.5 µM (7.5–12.2 µM), ∼720X higher than that for I_CaL_ (Figure 4E). The IC_50_ for hERG was 35 µM (23.4–52.4), ∼2,651X higher than that for I_CaL_ (Figure 4E).

### Intracellular recordings of human ventricular trabecular tissues

**FIGURE 5:**
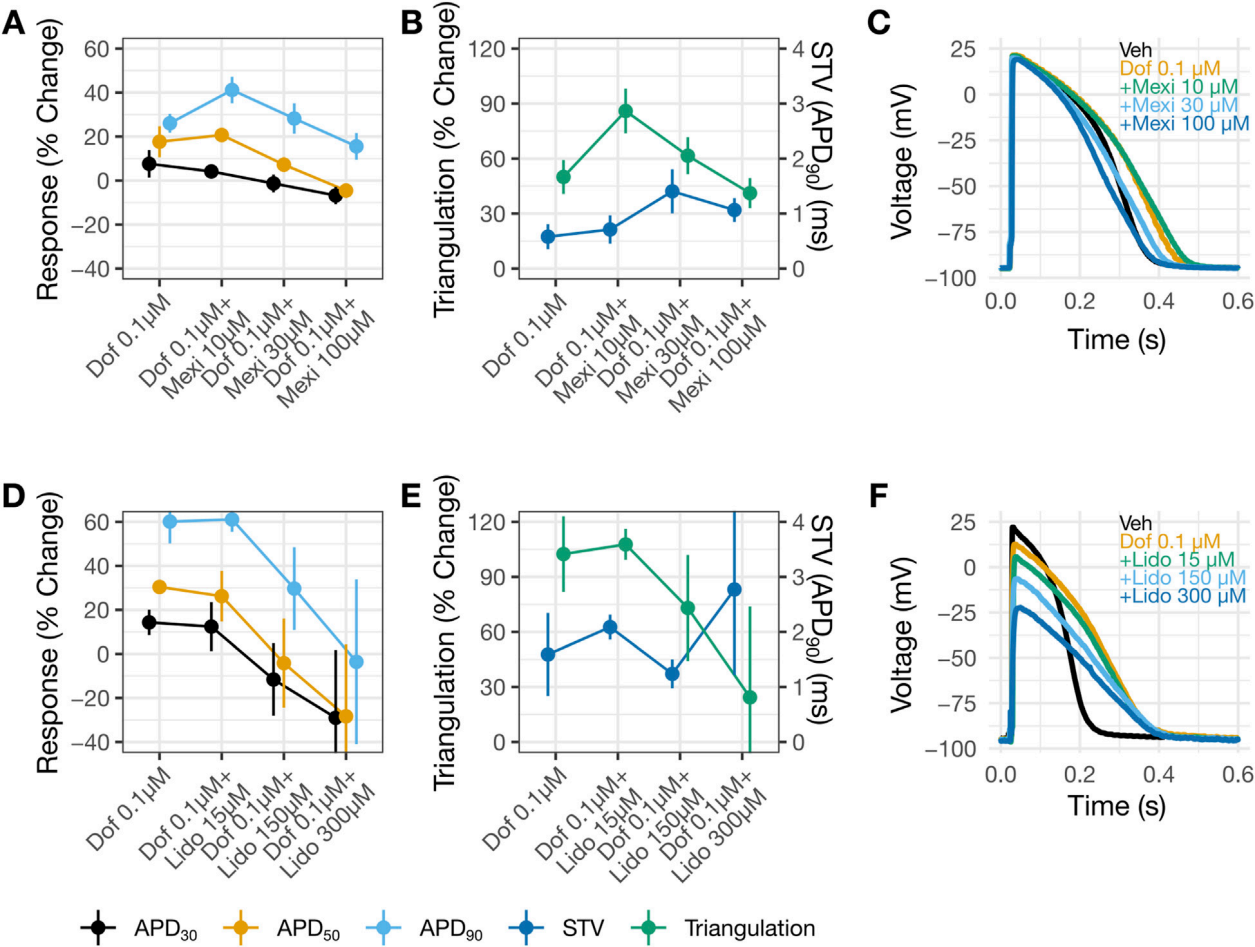
Shortening of dofetilide-induced APD prolongation by mexiletine or lidocaine. Changes from vehicle control recordings in APD parameters in panel **(A)** and in triangulation (left Y-axis) and STV (right Y-axis) in panel **(B)** for dofetilide with increasing concentrations of mexiletine. Panels **(D)** and **(E)** show similar plots for dofetilide and lidocaine. For panels **(A,B,D,E)**, data are presented as mean change from vehicle ±SE. Representative AP traces in **(C)** and **(F)** for vehicle, dofetilide, and dofetilide with increasing concentrations of mexiletine or lidocaine recorded at 1 Hz. DOF, Dofetilide; LIDO, lidocaine; MEX, mexiletine.

The consequence of applying dofetilide followed by mexiletine or lidocaine on ventricular AP characteristics was studied using human trabecular tissues. Dofetilide prolonged APD_30_, APD_50_, and APD_90_ (Figures 5A,C,D,F). The % APD prolongation was greatest for APD_90_, followed by APD_50_ then APD_30_, resulting in AP triangulation (Figures 5B,C,E,F). Application of 10 µM mexiletine resulted in further prolongation of APD_90_, without changes in APD_30_ or APD_50_ (Figures 5A,C), causing further AP triangulation (Figure 5B). In contrast, application of 15 µM lidocaine on top of dofetilide did not produce further changes in APD (Figure 5D) or further triangulation (Figures 5E,F). Subsequent application of higher concentrations of mexiletine (30 then 100 µM) and lidocaine (150 then 300 µM) led to concentration-dependent shortening of APD_30_, APD_50_, and APD_90_ (Figures 5A,C,D,F) and reduction of triangulation (Figures 5B,C,E,F). These findings are generally consistent with the reductions in the QT_C_ and J-T_peakC_ intervals by lidocaine and mexiletine (Figures 2A,B). In both sets of experiments, minimal changes were observed in STV (Figures 5B,E). For lidocaine, but not mexiletine, a reduction in AP height was observed (Figures 5C,F).

**FIGURE 6:**
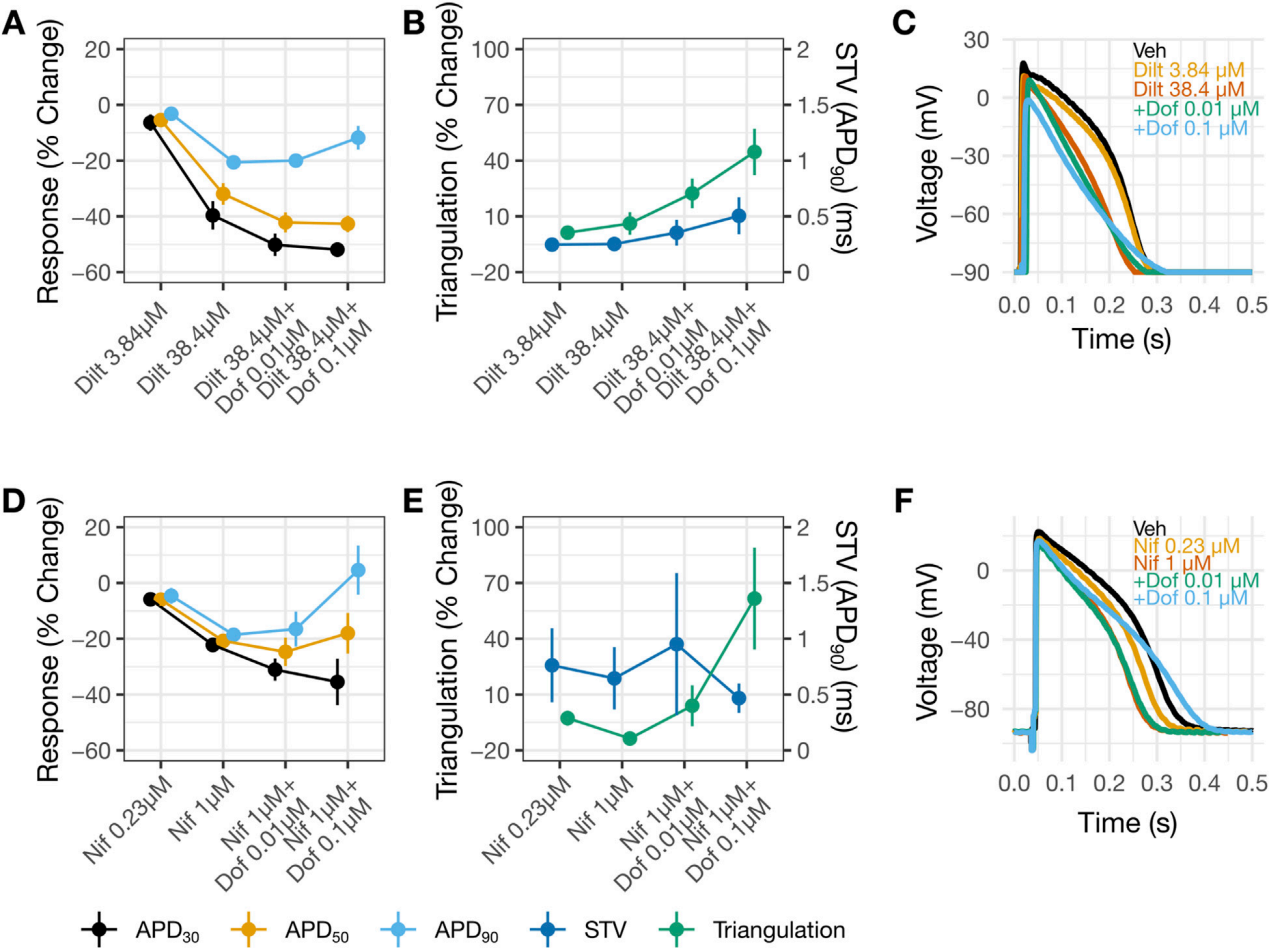
AP changes by diltiazem, but not nifedipine, suggest multi-ion channel block. Change from vehicle in panels **(A,B)** for diltiazem 38.4 µM with increasing concentrations of dofetilide and nifedipine 1 µM with increasing concentrations of dofetilide in panels **(D,E)**. In panels **(B,E)** the left y-axis corresponds to triangulation and the right y-axis correspond to STV. For panels **(A,B,D,E)** data is presented as mean change from vehicle and ±SE. Representative AP traces in **(C)** and **(F)** for vehicle, diltiazem or nifedipine with increasing concentrations of dofetilide at 1 Hz.

Another set of experiments examined the consequences of applying diltiazem (3.84 then 38.4 µM) or nifedipine (0.23 then 1 µM) followed by dofetilide (0.01 then 0.1 µM). Diltiazem (Figures 6A,C) or nifedipine (Figures 6D,F) caused concentration-dependent APD shortening. For diltiazem, the % reductions were greater in the earlier phases of the AP (i.e., APD_30_ and APD_50_), resulting in AP triangulation (Figures 6B,C). For nifedipine, the reductions were similar amongst APD_30_, APD_50_ and APD_90_ (Figures 6D,F), resulting in no AP triangulation (Figures 6E,F). Dofetilide at 0.01 µM was then added to 38.4 µM diltiazem (Figures 6A,C) or 1 µM nifedipine (Figures 6D,F). Recordings in these drug pairs showed further decreases in APD_30_ and APD_50_, whereas APD_90_ did not change. These changes suggest that the effects of 38.4 µM diltiazem or 1 µM nifedipine on myocytes continued to develop after 30 min of drug application, since dofetilide applied alone increased rather than decreased APD_30_ and APD_50_ (Figures 5A,C,D,F). Further increasing dofetilide to 0.1 µM led to an increase in APD_90_, but not APD_30_ or APD_50_. Dofetilide thus induced AP triangulation in the presence of diltiazem or nifedipine by preferentially affecting late repolarization. In both sets of experiments, minimal changes were observed in STV (Figures 5B,E).

All drug-induced changes in AP parameters are similar when the same tissue was paced at 1 and 2 Hz (Supplementary Figures S1, S2). Supplementary Figure S3 shows baseline AP characteristics, including APD_90_ and triangulation, for trabecula data included in this study combined to show data distribution and separated by donors. These distribution and values are consistent with the larger dataset published by the same authors (Page et al., 2016).

## Discussion

Na_V_1.5 and Ca_V_1.2 channels generate sustained inward currents that contribute to the ventricular APD hence are expected to contribute to the QT_C_ interval. Accordingly, inhibiting these currents should reduce the QT_C_ interval and mitigate hERG block-mediated QT_C_ prolongation. In the prospective clinical study by Johannesen et al., 2016 designed to test this hypothesis, concomitant administration of lidocaine or mexiletine shown to inhibit I_NaL_ in Crumb et al., 2016 shortened hERG block-mediated QT_C_ prolongation by dofetilide as hypothesized. However, diltiazem, demonstrated to inhibit Ba^2+^ current mediated by Ca_V_1.2 channels selectively in Crumb et al., 2016, failed to shorten hERG block-mediated QT_C_ prolongation by moxifloxacin. Diltiazem’s clinical results were difficult to reconcile considering the accepted role of Ca_V_1.2 channels in myocyte AP. Given that ion channel pharmacology results can be sensitive to experimental design and conduct, the present study reassessed inhibitory potencies on the hERG current, I_NaL_, and I_CaL_ for drugs evaluated in the clinical study plus nifedipine using more physiologically relevant protocols consistent with ICH S7B Q&A 2.1 best practice recommendations, and additionally evaluated these drugs’ impact on ventricular AP characteristics using human trabeculae tissue recordings. The new patch clamp results and AP recordings showed diltiazem to be a multi-ion channel blocker, not selective for Ca_V_1.2 channels as the prior study found. Across a concentration range of nearly four log units (Figure 4D), diltiazem shows concomitant inhibition for I_CaL_, I_NaL_, and importantly the hERG current. Thus, a plausible explanation for the lack of clinical QT_C_ shortening when diltiazem was co-administered with moxifloxacin is that diltiazem provides additional hERG inhibition which abolished its QT_C_-shortening effect mediated by I_CaL_ and I_NaL_ inhibition. The AP data also demonstrated that mexiletine or lidocaine reduced APD prolongation by dofetilide, consistent with the clinical results. Altogether, this study highlights the importance of generating ion channel data using physiologically relevant protocols and performing follow-up myocyte studies as described in ICH S7B to understand how multi-ion channel block alters cardiac APs and translates to drug-induced clinical ECG changes.

### The prior vs. present patch clamp results

Literature search shows that patch clamp studies from different publications or laboratories can exhibit large degrees of data variability. For the hERG current, dofetilide’s IC_50_s differed by ∼10X between the prior and present studies (Figure 1A vs. 4A). The reason for this difference is unclear. Both studies quantified drug effects at the repolarizing phase of the voltage command. Although the prior study evoked the current at 0.1 Hz while the present study used 0.2 Hz, stimulation frequency is not expected to impact dofetilide’s IC_50_ as this drug inhibits the hERG current in an use-dependent but frequency-independent manner (Tsujimae et al., 2004). Dofetilide, like other methanesulfonanilide compounds, becomes trapped within the closed hERG channels (Mitcheson et al., 2000) hence cannot dissociate regardless of the inter-pulse interval. As Kamiya et al., 2006 showed, once dofetilide block development occurred, repetitive pulses to hyperpolarized membrane potential did not cause recovery from block (see Figure 2C of Kamiya et al. 2006). In contrast, diltiazem’s IC_50_s on the hERG current were not different between the two studies, considering patch clamp data variability (Figure 1D vs. 4D). For the I_NaL_ experiments, the prior study used veratridine and quantified the steady state current evoked by the −15 mV step (see Figure 2A in Crumb et al., 2016); the present study used ATX-II and quantified the current at the voltage ramp down phase since the intent is to understand how inhibition of this inward current affects repolarization (Figure 3C). That both studies reported similar inhibitory potencies for lidocaine is surprising (Figure 1B vs. 4B). ATX-II binds to an extracellular site on Na^+^ channels, whereas veratridine binds to the transmembrane domain that overlaps sites where local anesthetics including lidocaine bind (for review, see (Ulbricht, 2005)) Consistently, this laboratory has previously demonstrated that lidocaine’s IC_50_ obtained in veratridine at room temperature and analyzed at the same region as Crumb et al., 2016 was 12.5X higher than that obtained in ATX-II (395.1 µM vs. 31.7 µM) (Wu et al., 2019). Mexiletine’s IC_50_s for I_NaL_ are not different between the prior and present studies (Figure 1C vs. 4C). This is consistent with Wu et al., 2019 which showed no impact of agonist (i.e., veratridine vs. ATX-II) on mexiletine inhibition of I_NaL_. Regarding Ca_V_1.2 channels, the prior study did not establish lidocaine’s IC_50_ to enable comparison. For mexiletine, the IC_50_ for the Ba^2+^ current in the prior study and that for I_CaL_ in the present study are interpreted as not different considering patch clamp data variability. For diltiazem, Crumb et al., 2016, reported an IC_50_ of 112.1 nM (Figure 1D), which is 11.6X more potent than the present result of 1.3 µM (Figure 4D). Previously this laboratory has reported that diltiazem’s IC_50_s on Ba^2+^ current through Ca_V_1.2 channels at ∼37 °C to be 0.8 µM, demonstrating that this drug’s block of Ca_V_1.2 channels is not impacted by the charge carrier (Ren et al., 2022). Ca_V_1.2 channel activity shows pronounced rundown in whole cell configuration whether Ba^2+^ or Ca^2+^ is used, and the rate of rundown is cell-dependent (Ren et al., 2022). During a pharmacology experiment, rundown would contribute additional current loss on top of the drug effect, leading to an apparent smaller IC_50_. Differences in laboratory-specific data acceptance criteria, including the rate of rundown tolerated for Ca_V_1.2 channel recordings, is thus a plausible explanation for the different IC_50_s for diltiazem between the prior and present study. Altogether, the prior and present results show that IC_50_s can differ in drug- and ionic current-specific manner, underscoring the challenge of using data generated from different laboratories using different experimental protocols and potentially different data acceptance criteria to predict clinical ECG change. Recently ICH S7B Q&A 2.1 was released to provide best practice recommendations for generating ion channel data for proarrhythmia risk assessment, and some elements presented include using as physiologically relevant protocols as feasible and practical to improve nonclinical-clinical translation (https://www.fda.gov/regulatory-information/search-fda-guidance-documents/e14-and-s7b-clinical-and-nonclinical-evaluation-qtqtc-interval-prolongation-and-proarrhythmic). Experimental design and conduct in the present study are consistent with ICH S7B Q&A 2.1 recommendations.

### Human trabecular AP recordings

The higher IC_50_ for diltiazem on I_CaL_ vs. hERG translates to a reduced selectivity for Ca_V_1.2 channels. The potency ratio for the hERG current and I_CaL_ based on the present results is 7, meaning that diltiazem can inhibit both inward and outward currents across a wide concentration range (Figure 4D). The AP results from human trabecular tissues corroborate the patch clamp results, showing greatest shortening of APD_30_, followed by APD_50_ then APD_90_ by diltiazem leading to AP triangulation–a pattern consistent with inhibition of I_NaL_ and I_CaL_ underlying AP plateau and hERG current involved in delayed repolarization (Figures 5A,B). In contrast, nifedipine, a selective Ca_V_1.2 channel blocker with a potency ratio for the hERG current and I_CaL_ ∼720 (Figure 4E), reduced APD_30_, APD_50_, and APD_90_ by a similar extent hence producing no concentration-dependent AP triangulation (Figures 5C,D). Applying dofetilide on top of diltiazem or nifedipine led to further AP triangulation, due to greater increase in APD_90_ than APD_50_ or APD_30_, consistent with the role of the hERG current in delayed repolarization.

When lidocaine or mexiletine were administered on top of dofetilide on the human trabecular tissue, concentration-dependent APD shortening was observed. These results are consistent with the clinical observation of QT_C_ and J-T_peakC_ shortening. Two observations indicate that these two drugs have distinct electrophysiology features in the heart. Firstly, an increase in triangulation was observed when the lower concentration of mexiletine (10 µM) was applied on top of dofetilide, due to further prolongation of APD_90_ but not APD_30_ or APD_50_ (Figures 4A,B), which was not observed when lidocaine was applied on top of dofetilide (Figures 5D,E). Secondly, concentration-dependent reduction in the AP height was observed for lidocaine but not for mexiletine (Figures 5C,F). In Johannesen et al., 2016, a similar magnitude of QT_C_ and J-T_peakC_ shortening was observed with co-administration of dofetilide and lidocaine or mexiletine, meaning no difference in the drug effects was observed clinically based on the analysis performed (Figure 2). Considering that ECG measurements reflect global signals and regional heterogeneity in cardiac ion channel expressions exist, changes in drug-induced changes in APD parameters may not always be observable at the ECG level. Regarding the AP height, the same laboratory that performed the present human trabecular tissue recordings has previously shown that some Na^+^ channel blockers reduced AP height (e.g., flecainide, lamotrigine, and mexiletine at 3X concentration as tested here) while ranolazine did not (see (Qu et al., 2017), Supplementary Material).

Triangulation of AP due to hERG block is proarrhythmic. This reflects a lengthening of the temporal window in the critical voltage range during which Ca_V_1.2 channels can recover from inactivation to generate an early afterdepolarization (EAD) – the cellular initiator of torsade. On the other hand, triangulation of AP due to block of Ca_V_1.2 and/or Na_V_1.5 channels in addition to hERG block may not be as proarrhythmic, since blocked Ca_V_1.2 channels cannot contribute to EAD. This view is simplistic, and it should be emphasized that: 1) torsade requires additional risk factors that compromises myocyte coupling; and 2) *the proportion of Ca*_*V*_ *1.2 and/or Na*_*V*_ *1.5 channels that needs to be blocked to reduce torsade risk remains to be defined.*

In this study, marginal changes in STV in response to drug exposures were observed. This is to be expected, given that the preparations used – ventricular trabecular tissues – are well electrically coupled, with baseline STV ranging between approximately 0.5–1.5 ms (Page et al., 2016; Qu et al., 2017). Drug-induced STV changes are typically small in ventricular trabecular tissues, and early afterdepolarizations (EADs) not frequently observed. In contrast, single ventricular myocytes have no coupling, and baseline STV values can range between 4 and 10 ms (Abi-Gerges et al., 2010; Ton et al., 2021). In the latter preparation, drugs can lead to larger changes in STV and cause EADs.

### Human induced pluripotent stem cell-derived cardiomyocytes (hiPSC-CMs)

This study used adult human ventricular tissues to assess drug-induced changes in AP parameters. HiPSC-CMs have been proposed as an alternative, though past studies have demonstrated that they do not fully replicate electrophysiological properties of native human myocardium, resulting in different changes relative to clinical findings in response to multi-ion channel block. Verapamil shows equal block potencies for hERG (IC_50_ ∼ 0.3 µM) (Alvarez Baron et al., 2025) and Ca_V_1.2 channels (IC_50_ ∼ 0.4 µM) (Ren et al., 2022). In clinical studies, it either did not alter the QT_C_ interval (Johannesen et al., 2014) or caused prolongation (Vicente et al., 2019). In human trabecular tissue recordings, verapamil did not alter APD_30_, APD_50_, or APD_90_ (Page et al., 2016). In hiPSC-CMs, verapamil caused concentration-dependent shortening in ΔΔAPD90_C_ and ΔΔFPD_C_ in VSD and MEA experiments, respectively (Blinova et al., 2017). When given with moxifloxacin, diltiazem also caused concentration-dependent shortening in ΔΔAPD90_C_ and ΔΔFPD_C_, which is in contrast with the clinical data summarized in Figure 2. Lidocaine and mexiletine, drugs that inhibit I_NaL_, were also co-applied with dofetilide, and the results show changes that depended on the cell source (Supplementary Figure S6 in (Blinova et al., 2017)). Increasing concentrations of lidocaine or mexiletine reduced dofetilide-induced increases ΔΔAPD90_C_ but not ΔΔFPD_C_ in hiPSC-CMs from Cell Dynamic International (now FUJIFILM; iCell). In contrast, either drug caused further increases in ΔΔAPD90_C_ and ΔΔFPD_C_ on top of dofetilide-induced prolongation in a concentration-dependent manner in hiPSC-CMs from Axiogenesis (now Ncardia; Cor.4U). Gene expression profiling showed that Ca_V_1.2 is overexpressed by 2.5X (Cor.4U) to 4X (iCell) on average in comparison to primary adult cardiac tissues, and Na_V_1.5 underexpressed, only ∼0.4X (Blinova et al., 2017). Regarding hERG, Cor.4U cells showed similar level of expression as adult human myocytes, while iCell showed 50% less. While integrative effect from hiPSC-CMs currently do not translate to clinical ECG changes, these cells could complement the traditional hERG assay to identify multi-ion channel blockers.

### Additional effects of Ca_V_1.2 channel blockers

The new patch clamp and AP recording results support the interpretation that diltiazem failed to shorten the QT_C_ interval when co-administered with moxifloxacin because it inhibited the hERG current in addition to I_CaL_ and I_NaL_ at clinical exposure achieved in Johannesen et al., 2016. Drugs that block Ca_V_1.2 channels can also cause PR prolongation. Diltiazem prolonged the PR interval by ∼20 ms. Previously this laboratory has reported that verapamil’s IC_50_ on I_CaL_ at the ramp current is 0.4 µM (Ren et al., 2022). In Johannesen et al., 2014, verapamil prolonged the PR interval by 32.1 ms. In contrast, nifedipine, a potent vasodilator, cannot be dosed in humans high enough to cause PR prolongation (for review, see (Prystowsky, 1988)). One explanation for the inconsistency between Ca_V_1.2 channel block and PR prolongation is these drugs have additional impact on the autonomic regulation, whether direct or indirect. Indeed, a slowing of AV node conduction and prolongation of the effective refractory period was observed for nifedipine using cardiac tissue from an animal model, leading Kawai and co-authors to conclude that reflexive sympathetic activation due to lowering of blood pressure obscured PR prolongation by this drug (Kawai et al., 1981). Diltiazem and verapamil reportedly have additional targets in the heart that affect autonomic regulation. A clinical study has found that diltiazem (but not nifedipine) depresses sympathetic activity similar to β-adrenergic blockers (Bekheit et al., 1990). Basic research showed that one effect may be mediated through parasympathetic activation which leads to activation of the K_ACh_ channels to hyperpolarize the myocyte membrane potential, as diltiazem’s ability to suppress epinephrine-induced arrhythmias was blocked by atropine, a muscarinic acetylcholine antagonist (Rabkin, 1992). Verapamil (but not diltiazem or nifedipine) binds to α-adrenergic receptors in the heart (Motulsky et al., 1983). Its active metabolite norverapamil binds to cardiac β-adrenergic receptors while verapamil itself shows weaker binding (Feldman et al., 1985). Finally, verapamil also binds to muscarinic acetylcholine receptors (Cavey et al., 1977). The additional mechanisms of Ca_V_1.2 channel blockers in the heart that indirectly affect electrophysiology need to be considered to understand these drugs’ different effects on cardiac function (Kawai et al., 1981; Mitchell et al., 1982; Prystowsky, 1988).

### Limitations and knowledge gaps

There are three limitations to this study. Firstly, this manuscript estimated drug block potencies using nominal drug concentrations, and not analytical concentrations. Drug concentrations can deviate from the nominal concentrations due to compound-specific factors and human errors (Alvarez Baron et al., 2022). The former include drug loss due to nonspecific binding to the patch clamp perfusion apparatus, potential insolubility at higher drug concentrations tested, and instability in the perfusion solution under the experimental condition. Human errors can also occur during the drug handling process. Concentration verification, if feasible, would improve drug potency estimations. A recent publication showed that the extent of loss for 28 drugs with different LogP values in the manual patch clamp rigs differed across five laboratories (Alvarez Baron et al., 2025). When comparing patch clamp data across studies, one should be mindful that the actual concentrations exposed to the recorded cells could be different. Secondly, stimulation frequencies used in the present study (0.1–0.2 Hz) were slower than the physiological heart rate range. Drug block of cardiac ion channels can be frequency-dependent (Weirich and Antoni, 1990; Nawrath and Wegener, 1997; Stork et al., 2007). For these drugs, estimating block at a lower stimulation rate would lead to underestimation of block potencies, thereby influencing nonclinical-to-clinical data translation. In fact, the same drug may not show the same frequency effect on all the channels. Thus, the multi-ion channel block profile as displayed in Figures 1, 4 is a construct of the experimental protocol. This means that the positions of the concentration-response curves for different currents relative to each other can change if the data were collected at a different stimulation frequency. Conducting experiments in the physiological heart rate range could further improve nonclinical-clinical translation. Finally, that I_NaL_ was recorded in the presence of ATX-II could have an impact on pharmacology. Using ATX-II was a trade-off to increase the signal-to-noise ratio because without this inducer, the sustained Na_V_1.5 current was simply too small to establish drug IC_50_s.

The hERG channels that mediate native I_Kr_ is comprised of hERG1a and hERG1b proteins (Jones et al., 2014). However, the present study, as well as the hERG assay performed by industry to support cardiac safety assessment, used/uses cell lines that express the hERG1a subunit only. hERG1a/1b channels exhibit larger current than hERG 1a channels, attributable to the heteromers’ increased activation rate and faster rate of recovery from inactivation in comparison to the homomers (Sale et al., 2008). Several studies have compared drug block potencies between the hERG1a and hERG1a/1b channels. Small-to-modest differences, ranging from 1.5X to 3X, were reported for E−4031 (Sale et al., 2008; Rios-Perez et al., 2021), dofetilide (Abi-Gerges et al., 2011; Rios-Perez et al., 2021), fluoxetine ((Abi-Gerges et al., 2011) but also see (Rios-Perez et al., 2021)), ebastine (Rios-Perez et al., 2021), and fentanyl (Tschirhart and Zhang, 2020). Majority of the drugs showed no pharmacological differences between these two channels, including five opioid agonists and antagonists–buprenorphine, norbuprenorphine, methadone, naloxone, and naltrexone (Tran et al., 2020) – and 47 of 50 drugs examined by Abi-Gerges et al., 2011 using an automated patch clamp platform (Abi-Gerges et al., 2011). For the latter study, the authors shared that longitudinal assessment of cisapride by the same laboratory showed variations in block potency of ± 2X, not much lower than the block potency differences observed for some drugs. This piece of information is important as it informs the likelihood that observed differences in IC_50_s reflect natural data distribution if the laboratory were to test the same drug on the same channels repeatedly (i.e., assay reproducibility), or genuine differences in pharmacology. A recent paper resulting from a Health & Environmental Science Institute (HESI)-coordinated multiple laboratory effort showed that the manual patch clamp hERG assay reproducibility was ∼5X (Alvarez Baron et al., 2025), in the same range as that reported by Abi-Gerges et al., 2011. Assay reproducibility on other cardiac current are likely different, and the same HESI authors are currently evaluating assay reproducibility for I_CaL_, I_NaL_, and peak Na^+^ current or I_NaP_ experiments (see HESI BAA Patch Clamp Ion Channel Study at https://hesiglobal.org/cardiac-safety/). Regarding AP recordings in trabecular tissues, the same laboratory that generated data for the present study had also tested dofetilide previously (Page et al., 2016; Qu et al., 2017). In 25 experiments conducted on different occasions using tissues of 11 donors, the average percent change in APD_90_ by 100 nM dofetilide was 80.1%, with upper and lower bounds of the associated 95% CI being 53.4% and 106.7%. Continuing to build such knowledge base regarding assay reproducibility will pay dividends in the long run to promote further use of nonclinical data to interpret and predict drug-induced changes in cardiac electrophysiology.

## Conclusion

Patch clamp assessment of drug block potencies and AP recordings in human myocytes are the core and follow-up assays in ICH S7B. The results of these assays depend on the experimental protocol used and are associated with uncertainty that needs to be considered when drawing study conclusions. This study shows the importance of using physiologically relevant protocols to generate ion channel pharmacology data to improve nonclinical-clinical translation and the utility of these assays in assessing the consequences of multi-ion channel block on cardiac electrophysiology.

## Data Availability

The datasets presented in this study can be found in online repositories. The names of the repository/repositories and accession number(s) can be found below: https://osf.io/69ght/.

## Supplementary material

SUPPLEMENTARY FIGURE S1 Similar drug effects on AP characteristics with 1 and 2 Hz pacing rate: Dofetilide plus diltiazem or nifedipine. Changes from vehicle control recordings in APD parameters in panel **(A)** and in triangulation (left Y-axis) and STV (right Y-axis) in panel **(B)** for diltiazem with increasing concentrations of dofetilide. Panels **(C,D)** show similar plots for nifedipine and dofetilide. For all panels, data are presented as mean change from vehicle ±SE. DIL, diltiazem; DOF, Dofetilide; NIF, nifedipine.

SUPPLEMENTARY FIGURE S2 Sotalol plus diltiazem and moxifloxacin plus diltiazem. Changes from vehicle control recordings in APD parameters in panel **(A)** and in triangulation (left Y-axis) and STV (right Y-axis) in panel **(B)** for diltiazem with increasing concentrations of sotalol. Panels **(C,D)** show similar plots for diltiazem and moxifloxacin. For all panels, data are presented as mean change from vehicle ±SE. DIL, diltiazem; SOT, Sotalol; MOXI, Moxifloxacin.

SUPPLEMENTARY FIGURE S3 Baseline characteristics of APD_90_ and triangulation at 1 Hz within and across donors. Distribution of baseline measurements within each donor at 1 Hz for APD_90_ in panel **(A)** and triangulation in panel **(B)**. Panels **(C,D)** show the distribution across donors of APD_90_ and triangulation, respectively.
