# Supplementary figures and images for: Integrating drug effects on individual cardiac ionic currents and cardiac action potentials to understand nonclinical translation to clinical ECG changes
Source: Front Pharmacol. 2025 Nov 10;16:1674861. doi: 10.3389/fphar.2025.1674861 (PMC12640937; doi:10.3389/fphar.2025.1674861)

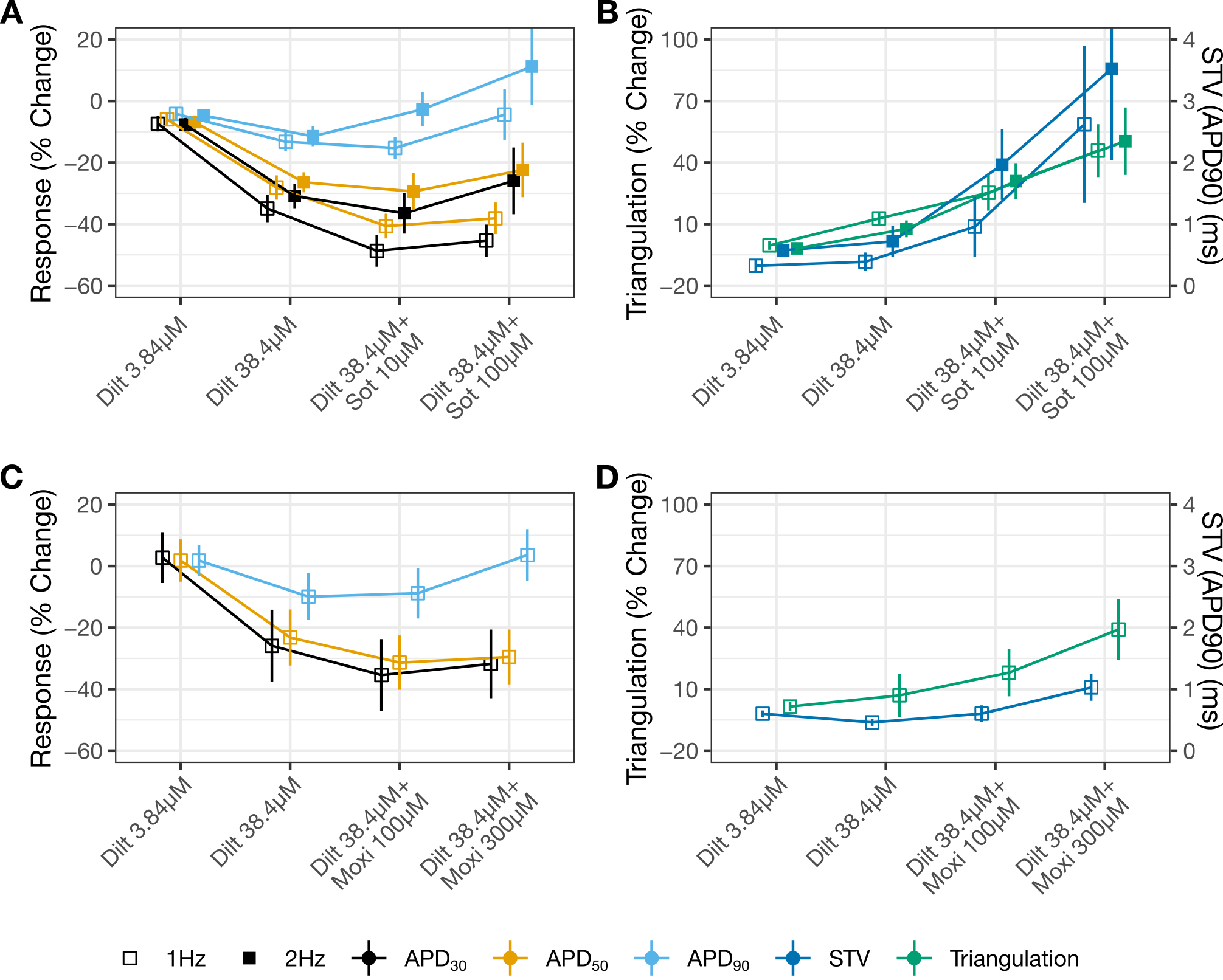

Supplement: Supplementary file 1 [file Image3.tiff]

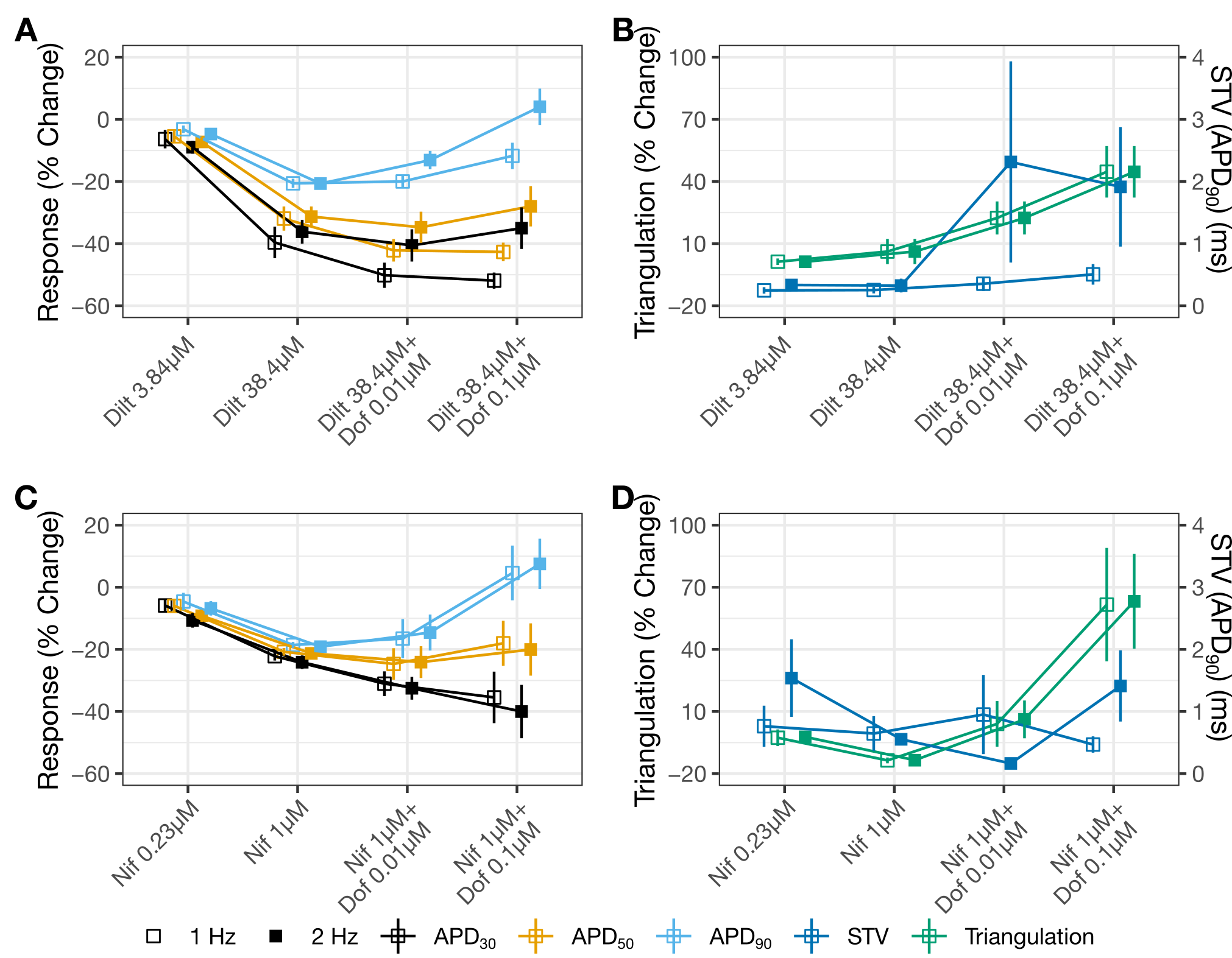

Supplement: Supplementary file 2 [file Image1.tiff]

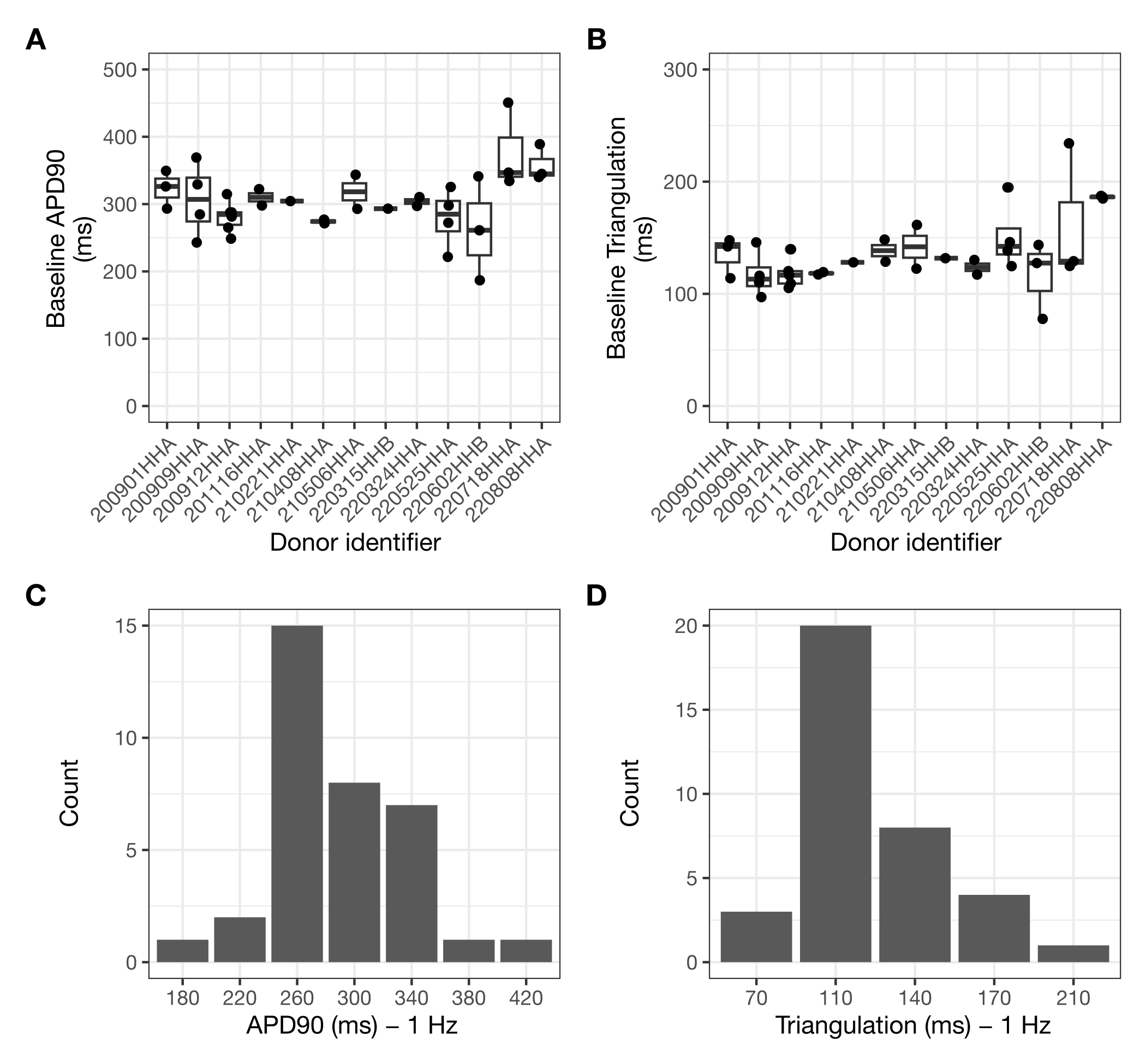

Supplement: Supplementary file 4 [file Image2.tiff]
