# Supplementary material for: Integrating drug effects on individual cardiac ionic currents and cardiac action potentials to understand nonclinical translation to clinical ECG changes
Source: Front Pharmacol. 2025 Nov 10;16:1674861. doi: 10.3389/fphar.2025.1674861 (PMC12640937; doi:10.3389/fphar.2025.1674861)
Supplement: Supplementary file 3 [file Table1.docx]

Supplementary Table 1: Additional experiments performed using adult human primary ventricular trabeculae not presented in the main text.

Data were not collected at 2 Hz for diltiazem and moxifloxacin.
